# Supplementary material for: Rural infrastructure and poverty in China
Source: PLoS One. 2022 Jun 9;17(6):e0266528. doi: 10.1371/journal.pone.0266528 (PMC9182566; doi:10.1371/journal.pone.0266528)
Supplement: Attachment — Submitted filename: Response to Reviewers.docx [file pone.0266528.s003.docx]

**Response Letter**

Dear Editors and Reviewers,

We appreciate the opportunity to modify our manuscript according to the critical comments concerning our manuscript entitled “*Rural Infrastructure and Poverty in China*” (Manuscript Number: PONE-D-22-00776). We would like to thank the editors and the review team for your constructive comments and suggestions on our paper. They are all valuable and very helpful for revising and improving our paper, and simultaneously they are of great significance to give instructions to our research. In the stage of Minor Revision, we have also studied the comments carefully and made revisions which we hope to meet with approval. With a concerted effort to address all of the valuable advice, **we have revised the paper to a more accurate and concise style including reference list and the format of the main text.** The manuscript PDF is compiled from the LaTeX files provided by PLOS.

Also, it should be noted that we highlight the important changes **in yellow** in our revised manuscript. Below, we provide our detailed responses in tabular form to explain how your points have been included in the revision. As you will see, we have made every possible attempt to address your concerns, and we hope you will find our revision acceptable. Once again, thank you for helping us to improve the manuscript greatly.

**Responses to Journal Requirements**

| **Comments** | **Responses** |
| --- | --- |
| 1. If you would like to make changes to your financial disclosure, please include your updated statement in your cover letter. Guidelines for resubmitting your figure files are available below the reviewer comments at the end of this letter. | Thanks for the reminder. We have made changes to our financial disclosure, which can be seen in our cover letter. |
| 2. Please review your reference list to ensure that it is complete and correct. If you have cited papers that have been retracted, please include the rationale for doing so in the manuscript text, or remove these references and replace them with relevant current references. Any changes to the reference list should be mentioned in the rebuttal letter that accompanies your revised manuscript. If you need to cite a retracted article, indicate the article’s retracted status in the References list and also include a citation and full reference for the retraction notice. | Thanks a lot for the reminder. We have reviewed our reference list to ensure that it is complete and correct and revised the reference style as outlined in the ICMJE sample references.  Besides, we replace the 7^th^ reference “Fan, S., Nyange, D., Rao, N.” with “Hruschka D J, Hadley C, Hackman J.”, as the latter can better explain the Trickle-down effect, which is listed as following.  Hruschka D J, Hadley C, Hackman J. Material wealth in 3D: mapping multiple paths to prosperity in low-and middle-income countries. PloS one. 2017;12(9): e0184616. |

**Responses to Reviewers**

| **Comments** | **Responses** |
| --- | --- |
| Reviewer #1: All problems have been addressed. It is recommended for publication.  Reviewer #2: The authors have revised their manuscript based on the review comments.  However, the format of the main text should be coherent with the requirement of Plos One. | Thank you very much for your appreciating the manuscript. As you suggested, we have used the LaTeX template provided by PLOS and compiled the LaTeX files as PDF to ensure the format of the main text should be coherent with the requirement of Plos One.  Besides, we have revised the reference style as outlined in the ICMJE sample references, which is required by Plos One. |
